# Hyperacute Cytokine Kinetics and Early Interleukin-10 Elevation as Predictors of Neurological Outcome in Post-Cardiac Arrest Syndrome

**DOI:** 10.3390/jcm15166376

**Published:** 2026-08-18

**Authors:** Jea Hun Oh, Hyo Joon Kim, Kyung Man Cha, Daehee Kim, Kiwook Kim, In Soo Kim, Ji Hoon Kim, Chun Song Youn, Sang Hoon Oh, Hyo Jin Bang, Ae Kyung Gong, Ji Sook Lee

**Affiliations:** 1 Department of Emergency Medicine, Eunpyeong St. Mary’s Hospital, College of Medicine, The Catholic University of Korea, Seoul 06591, Republic of Korea; emojh81@gmail.com; 2 Department of Emergency Medicine, Seoul St. Mary’s Hospital, College of Medicine, The Catholic University of Korea, Seoul 06591, Republic of Korea; ycs1005@catholic.ac.kr (C.S.Y.);; 3 Department of Emergency Medicine, St. Vincent’s Hospital, College of Medicine, The Catholic University of Korea, Seoul 06591, Republic of Korea; cha.kyungman@gmail.com; 4 Department of Emergency Medicine, Incheon St. Mary’s Hospital, The Catholic University of Korea, Seoul 06591, Republic of Korea; md.kim.daehee@gmail.com; 5 Department of Emergency Medicine, Uijeongbu St. Mary’s Hospital, College of Medicine, The Catholic University of Korea, Seoul 06591, Republic of Korea; kiwookkim@catholic.ac.kr; 6 Department of Emergency Medicine, Daejeon St. Mary’s Hospital, College of Medicine, The Catholic University of Korea, Seoul 06591, Republic of Korea; kkis329@naver.com; 7 Department of Emergency Medicine, Bucheon St. Mary’s Hospital, College of Medicine, The Catholic University of Korea, Seoul 06591, Republic of Korea; intimator@naver.com

**Keywords:** cardiac arrest, cytokines, IL-10, immunoparalysis, neurological outcome, post-cardiac arrest syndrome, targeted temperature management

## Abstract

**Background:** Post-cardiac arrest syndrome (PCAS) involves a complex interplay between systemic inflammatory and compensatory anti-inflammatory responses. The hyperacute kinetics of these cytokines and their incremental prognostic value beyond established brain injury biomarkers remain inadequately characterized. We aimed to evaluate whether the 6 h post–return-of-spontaneous-circulation (ROSC) cytokine profile predicts neurological outcome, with the Th1/Th2 ratio largely reflecting the IL-10 signal. **Methods:** This retrospective analysis of prospectively collected data from a single-center cardiac arrest registry included 56 cardiac arrest survivors (40 out-of-hospital, 16 in-hospital) treated with targeted temperature management (TTM) at 33 °C or 36 °C between January 2024 and December 2025. Serum IL-6, IL-10, IFN-γ (interferon-γ), and TNF-α (tumor necrosis factor-α) were measured at five time points (initial presentation (INIT), and 6, 24, 48, and 72 h post-ROSC). Neurological outcome was assessed using the Cerebral Performance Category (CPC) scale at 6 months after cardiac arrest, with poor outcome defined as CPC 3–5. **Results:** Eighteen patients (32.1%) had a good outcome (CPC 1–2) and 38 (67.9%) had a poor outcome at 6 months after cardiac arrest. Among the 38 poor-outcome patients, 2 were classified as CPC 3, 8 as CPC 4, and 28 as CPC 5 (death) at 6 months; the 6-month outcome was available for all 56 patients, with no loss to follow-up. Patients with poor outcomes exhibited a synchronized surge of IL-6 and IL-10 peaking at 6 h post-ROSC. IL-10 at 6 h showed the highest discriminative power among cytokines (area under the curve (AUC) 0.844) compared with IL-6 (AUC 0.761). Multivariable analysis using Youden-derived cut-offs revealed that IL-10 ≥ 154.32 pg/mL (adjusted odds ratio (aOR) 15.28, 95% CI 2.02–115.38, *p* = 0.008) and a Th1/Th2 ratio ≥ 0.0314 (aOR 0.09, 95% CI 0.01–0.61, *p* = 0.013) were independently associated with neurological outcome after adjustment for age and initial shockable rhythm. **Conclusions:** The 6 h post-ROSC window is a critical inflection point for immune dysregulation in PCAS. Early IL-10 elevation and collapse of the Th1/Th2 balance are independently associated with poor neurological recovery and add incremental prognostic information to established brain injury biomarkers. External validation in larger prospective cohorts is required.

## 1. Introduction

Post-cardiac arrest syndrome (PCAS) is characterized by a sepsis-like systemic ischemia–reperfusion injury following the return of spontaneous circulation (ROSC) [1,2]. This process triggers an immediate release of pro-inflammatory cytokines and a simultaneous activation of anti-inflammatory pathways. The severity of subsequent secondary brain injury is largely dictated by the balance between these competing immune responses during the hyperacute phase [3].

Recent studies have highlighted the role of cellular immunity and innate effector cells in neuroinflammation. Pro-inflammatory T cells (Th1/Th17) rapidly infiltrate the brain parenchyma through a disrupted blood–brain barrier [4], whereas Th2 cells and regulatory T cells secrete interleukin-10 (IL-10) to modulate this response [5]. However, an exaggerated compensatory anti-inflammatory response can lead to an early immunoparalysis-like state, thereby exacerbating neuronal damage and increasing susceptibility to secondary infections [6,7].

While early simultaneous pro- and anti-inflammatory activation has been reported at hospital admission [1], the precise time-course kinetics during the first hours after ROSC remain poorly defined. Most prior studies relied on broad intervals (e.g., day 1 versus day 3) that may obscure the critical immunological inflection point [8]. A recent single-cell study characterized a hyperacute cytokine and immune-checkpoint axis after cardiac arrest [9]; however, it did not assess the incremental prognostic value of individual early cytokines over established brain-injury biomarkers, which is the focus of the present study. Furthermore, the incremental prognostic value of early cytokines beyond established brain injury biomarkers, such as neuron-specific enolase (NSE) and S100B [10], has not been rigorously evaluated.

We therefore investigated the sequential kinetics of cytokines in cardiac arrest survivors undergoing TTM and evaluated whether the 6 h Th1/Th2 balance is independently associated with neurological outcomes and provides incremental predictive value over standard brain injury biomarkers.

## 2. Materials and Methods

### 2.1. Study Design and Setting

This was a retrospective analysis of data prospectively collected in a single-center cardiac arrest registry at a tertiary academic emergency medical center. Adult patients (≥18 years) admitted following successful resuscitation from cardiac arrest, who remained comatose, and were treated with TTM between January 2024 and December 2025 were considered for inclusion. Although the Act on Decisions on Life-Sustaining Treatment has permitted the withdrawal of life-sustaining therapy in Korea since 2018, under our institutional policy, such withdrawal is undertaken only for organ donation. Because no organ donation occurred during the study period, no patient underwent withdrawal of life-sustaining therapy, and all deaths resulted from medical causes. This study was conducted in accordance with the Declaration of Helsinki and approved by the Institutional Review Board of Seoul St. Mary’s Hospital (protocol code KC19OESI0191; date of initial approval 4 June 2019, currently valid through 3 June 2027 under annual continuing review of the prospective registry). Written informed consent was obtained from the legal surrogates of all patients.

### 2.2. Study Population

We included consecutive adult survivors of either out-of-hospital cardiac arrest (OHCA) or in-hospital cardiac arrest (IHCA) who achieved sustained ROSC (≥20 min) and who underwent TTM. Exclusion criteria were (i) age < 18 years; (ii) traumatic etiology of arrest; (iii) terminal malignancy or pre-existing CPC ≥ 3; (iv) pregnancy; and (v) absence of cytokine measurement at the 6 h time point.

### 2.3. Targeted Temperature Management Protocol

TTM was initiated as soon as feasible after ROSC, according to institutional protocol. The target temperature (33 °C or 36 °C) was selected by the treating intensivist based on patient characteristics and contraindications. The TTM protocol reflected the guidelines in effect during the enrolment period; we acknowledge the subsequent shift toward normothermia and active fever avoidance (e.g., the TTM2 trial) and note that the early cytokine kinetics examined here are unlikely to be specific to the temperature target. Cooling was achieved using surface or endovascular devices and maintained for at least 24 h, followed by controlled rewarming at 0.25 °C/h. Standard post-resuscitation care, including hemodynamic optimization, mechanical ventilation, sedation, and seizure prophylaxis, followed contemporary international guidelines [11,12].

### 2.4. Sample Collection and Cytokine Measurement

Peripheral venous blood samples were drawn at five pre-specified time points: at initial presentation (INIT, defined as the first sample obtained at emergency department arrival or within 1 h of ROSC for in-hospital cases), and at 6, 24, 48, and 72 h after sustained ROSC. Samples were centrifuged within 30 min of collection, and serum was aliquoted and stored at −80 °C until batched analysis. Serum concentrations of IL-6, IL-10, IFN-γ, and TNF-α were quantified using a multiplex bead-based immunoassay (MILLIPLEX^®^ MAP Human Cytokine/Chemokine/Growth Factor Panel A, Cat. No. HCYTA-60K; Merck Millipore, Burlington, MA, USA) according to the manufacturer’s instructions. For this panel, the manufacturer-specified intra- and inter-assay coefficients of variation were <15% and <20%, respectively, and the minimum detectable concentrations for the analyzed cytokines were 0.14 pg/mL (IL-6), 0.91 pg/mL (IL-10), 0.86 pg/mL (IFN-γ), and 5.39 pg/mL (TNF-α). Serum S100B was measured with the Elecsys^®^ S100 immunoassay (Roche Diagnostics, Mannheim, Germany) on a Cobas e411 analyzer, and serum neuron-specific enolase (NSE) was measured with the Roche electrochemiluminescence method (NSE, Cobas e601; Roche Diagnostics, Mannheim, Germany). According to the manufacturer, the intra- and inter-assay coefficients of variation for the cytokine panel (MILLIPLEX Human Cytokine/Chemokine/Growth Factor Panel A, Cat. no. HCYTA-60K) were <15% and <20%, respectively; analyte-specific lower limits of quantification were those specified in the lot-specific certificate of analysis, and measured IFN-γ concentrations (minimum 0.98 pg/mL) lay within the quantifiable range rather than at a detection floor. Values exceeding the upper limit of detection (>10,000 pg/mL for IL-6) were assigned the upper limit value for analysis (right-censored treatment); a sensitivity analysis excluding these values was performed and yielded a consistent direction of effect (Supplementary Material). Because the excluded above-range IL-6 values were predominantly from poor-outcome patients, this exclusion biases conservatively against, rather than inflates, the observed association.

### 2.5. Definition of Outcomes

The primary outcome was poor neurological recovery, defined as a Cerebral Performance Category (CPC) score of 3–5 at 6 months after cardiac arrest. Good outcome was defined as CPC 1–2. The 6-month CPC was ascertained by a research coordinator through telephone follow-up 6 months after cardiac arrest. CPC was assessed prospectively by the treating clinical team and verified retrospectively from the medical record by two trained investigators blinded to cytokine results; disagreements were resolved by consensus.

### 2.6. Statistical Analysis

Continuous variables are presented as median (interquartile range, IQR) and categorical variables as number (percentage). Between-group comparisons used the Mann–Whitney U test for continuous variables and Fisher’s exact test for categorical variables, as appropriate. The Th1/Th2 ratio was calculated as IFN-γ divided by IL-10 at the same time point as a surrogate marker of T-helper polarization. Because IFN-γ did not differ significantly between outcome groups (see Results), the Th1/Th2 ratio is not statistically independent of IL-10; the two are therefore interpreted as related rather than as separate lines of evidence. Multiple comparisons across cytokines and time points were not formally corrected because the primary analyses were pre-specified at the 6 h time point; exploratory comparisons at other time points are reported with unadjusted *p*-values and should be interpreted descriptively.

Receiver operating characteristic (ROC) curve analysis was used to evaluate the discriminative ability of biomarkers for poor outcome at 6 h, and optimal cut-off values were determined using the Youden index. As a post hoc, exploratory analysis, correlations between IL-10 and leukocyte subset counts were assessed with Spearman’s rank correlation coefficient. Multivariable logistic regression was performed to assess independent associations of biomarkers with outcome, adjusting for age (continuous) and initial shockable rhythm (binary), which were chosen a priori based on clinical importance and the events-per-variable consideration. Based on the smaller outcome group (18 events), this corresponds to 6.0 events per variable, below the conventional threshold of 10, which motivated the use of Firth penalized-likelihood regression. Two separate models were fitted for IL-10 and Th1/Th2 ratio to avoid collinearity given that both share IL-10.

Three sensitivity analyses were performed: (i) restricting the cohort to OHCA cases (n = 40); (ii) including time to ROSC as an additional covariate; and (iii) replacing dichotomized cut-offs with log-transformed continuous values. The incremental predictive value of IL-10 at 6 h over established brain injury biomarkers (S100B at 6 h and NSE at 48 h) was assessed using likelihood-ratio (LR) tests of nested logistic regression models, with IL-10 entered as a log10-transformed continuous variable and each comparison restricted to complete cases for the respective reference marker.

To address the risk of quasi-complete separation and small-sample bias in the multivariable models, both models were additionally re-estimated using Firth’s penalized-likelihood logistic regression [13], with confidence intervals based on the profile penalized likelihood. Optimism of the apparent discrimination was quantified by bootstrap internal validation [14] (Harrell’s optimism-correction method, 1000 resamples), and optimism-corrected areas under the curve (AUCs) are reported alongside apparent values; repeated five-fold cross-validation was used as a secondary estimate. Statistical significance was set at two-sided *p* < 0.05. Analyses were performed using Python (version 3.12; pandas, scipy, statsmodels, and scikit-learn libraries) and R (version 4.3; logistf and pROC packages). This study was reported in accordance with the STROBE statement for observational studies [15] (Supplementary Checklist S1).

## 3. Results

### 3.1. Baseline Characteristics

**Figure 1:**
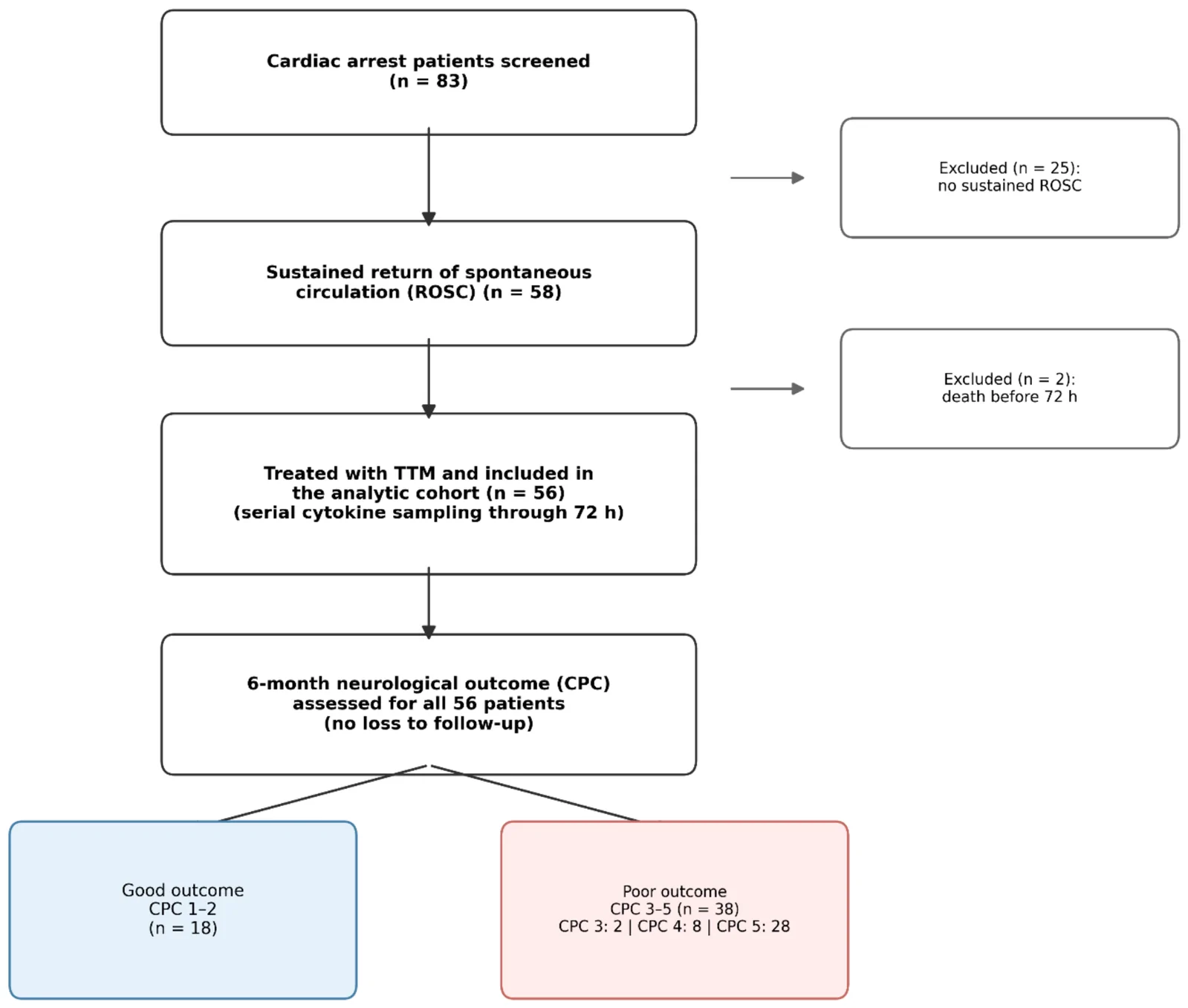
Participant flow diagram. Of 83 cardiac arrest patients screened, 58 achieved sustained return of spontaneous circulation and 56 were treated with targeted temperature management and included in the analytic cohort. Twenty-five patients were excluded for absence of sustained ROSC and two for death before 72 h. Six-month Cerebral Performance Category was available for all 56 patients (no loss to follow-up).

Of the 56 patients (40 OHCA, 16 IHCA), 18 (32.1%) had a good outcome (CPC 1–2) and 38 (67.9%) had a poor outcome (CPC 3–5) at 6 months after cardiac arrest. The participant flow is summarized in Figure 1. Patients with a good outcome were more likely to have an initial shockable rhythm (66.7% vs. 13.2%, *p* < 0.001), a cardiac etiology (94.4% vs. 55.3%, *p* = 0.005), and a shorter time to ROSC (median 19.5 vs. 33.0 min, *p* = 0.011). Age, sex, comorbidities, and the proportion treated at a target temperature of 33 °C did not significantly differ between groups (Table 1). 

### 3.2. Time Course of Cytokines

**Figure 2:**
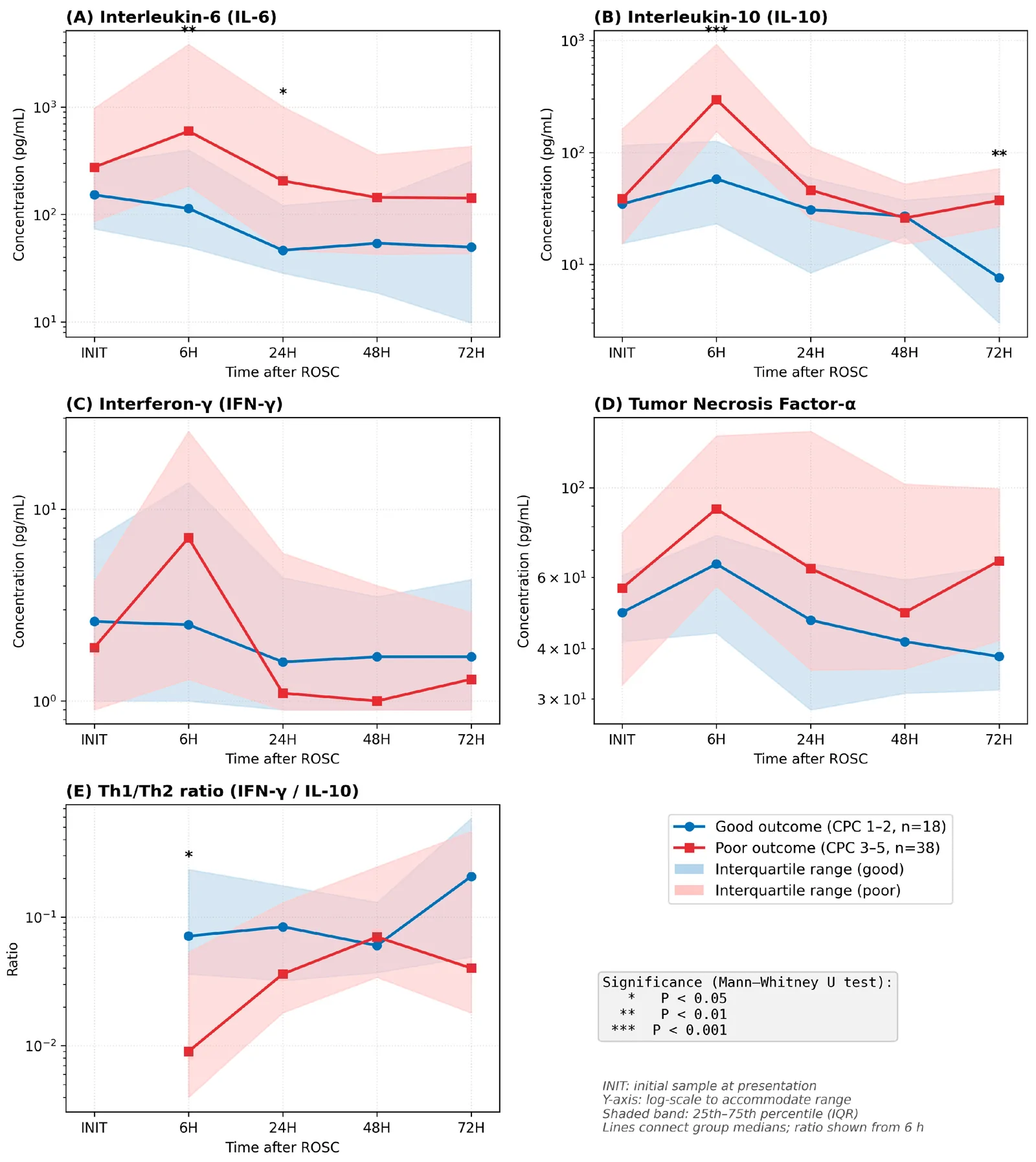
Hyperacute time course of cytokines and the Th1/Th2 ratio according to neurological outcome. Temporal dynamics of serum concentrations of (**A**) IL-6, (**B**) IL-10, (**C**) IFN-γ, and (**D**) TNF-α from initial presentation (INIT) up to 72 h after ROSC. Panel (**E**) shows the trajectory of the Th1/Th2 ratio (IFN-γ/IL-10). The y-axes are presented on a logarithmic (log10) scale. Blue lines indicate patients with a good outcome (CPC 1–2, n = 18); red lines indicate poor outcome (CPC 3–5, n = 38). Shaded bands indicate the interquartile range. The poor-outcome group exhibits a synchronized surge of IL-6 and IL-10 peaking at 6 h, accompanied by an early collapse of the Th1/Th2 ratio.

A synchronized surge of IL-6 and IL-10 was observed at 6 h post-ROSC in the poor-outcome group (Table 2; Figure 2). Median IL-10 at 6 h was significantly higher in the poor-outcome group (296.8 [154.3–920.1] vs. 57.9 [23.1–125.8] pg/mL, *p* < 0.001), as was IL-6 (596.2 [185.8–3813.5] vs. 113.8 [49.9–398.7] pg/mL, *p* = 0.004). The Th1-signature cytokine IFN-γ and pro-inflammatory TNF-α did not differ significantly between groups at 6 h (*p* = 0.403 and *p* = 0.063, respectively). A delayed second elevation of IL-10 was observed at 72 h in the poor-outcome group (37.3 vs. 7.6 pg/mL, *p* = 0.009).

### 3.3. ROC Analysis and Multivariable Logistic Regression

**Figure 3:**
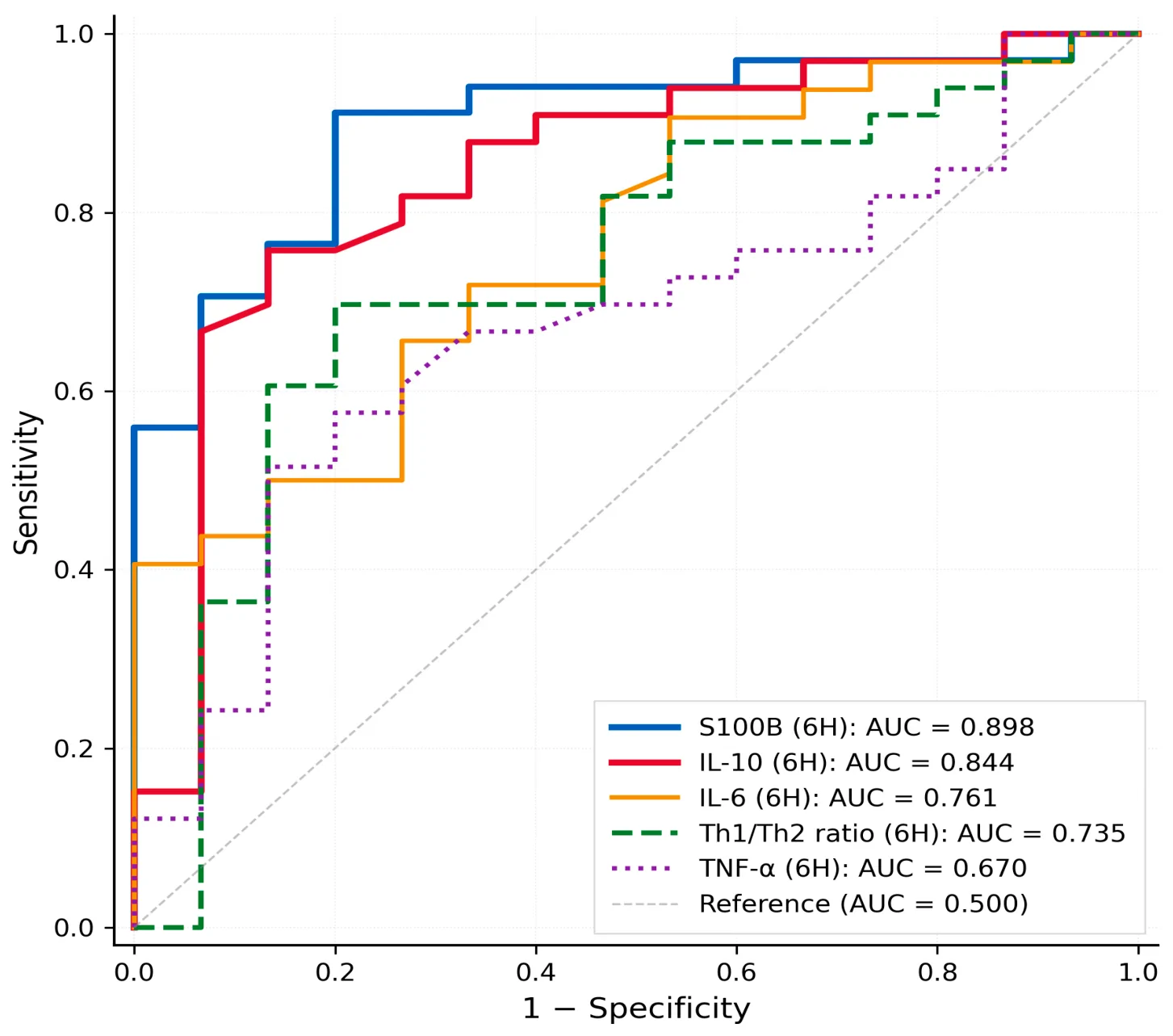
Receiver operating characteristic (ROC) curves for predicting poor neurological outcome (Cerebral Performance Category 3–5) using biomarkers measured at 6 h after return of spontaneous circulation (ROSC). Interleukin-10 (IL-10) demonstrated excellent discriminative power (area under the curve [AUC] = 0.844). The Th1/Th2 ratio (IFN-γ/IL-10) showed acceptable predictive value (AUC = 0.735). For comparison, S100B at 6 h is shown (AUC = 0.898).

At 6 h post-ROSC, IL-10 demonstrated the highest discriminative power among the four cytokines for poor neurological outcome (AUC 0.844), exceeding IL-6 (AUC 0.761), TNF-α (AUC 0.670), and IFN-γ (AUC 0.577); the whole-cohort Th1/Th2 ratio yielded an AUC of 0.735, and the ROC curves for the better-performing markers are shown in Figure 3. Optimal cut-offs derived from the Youden index were 154.32 pg/mL for IL-10 and 0.0314 for the Th1/Th2 ratio. Given the 24 unadjusted comparisons in Table 2, only the 6 h IL-10 difference remained significant after Bonferroni correction; the 72 h IL-10 rise and the 6 h Th1/Th2 difference are therefore regarded as exploratory. In multivariable logistic regression adjusting for age and initial shockable rhythm, IL-10 ≥ 154.32 pg/mL (aOR 15.28, 95% CI 2.02–115.38; *p* = 0.008) and a Th1/Th2 ratio ≥ 0.0314 (aOR 0.09, 95% CI 0.01–0.61; *p* = 0.013, indicating that a preserved ratio was protective) were independently associated with neurological outcome (Table 3).

### 3.4. Sensitivity Analyses

In the OHCA-only subgroup (n = 40, 10 good and 30 poor outcomes), IL-10 at 6 h retained excellent discrimination (AUC 0.896; median 29.3 vs. 214.1 pg/mL for good vs. poor, *p* < 0.001), and the Th1/Th2 ratio retained acceptable discrimination (AUC 0.753). Direction and magnitude of associations were consistent with the primary analysis. Adjustment for time to ROSC did not materially alter the associations of IL-10 or the Th1/Th2 ratio with outcome (data shown in Supplementary Table S1). Replacing dichotomized cut-offs with log-transformed continuous values yielded consistent inference.

### 3.5. Incremental Value over Established Brain Injury Biomarkers

S100B at 6 h and NSE at 48 h were themselves strongly associated with poor outcome, with marginal AUCs of 0.898 and 0.872, respectively, on all patients in whom the respective marker was available (Figure 3). Incremental value was assessed by adding log-transformed IL-10 at 6 h to nested logistic models within the complete-case subsets in which both the reference marker and IL-10 were available (n = 43 for S100B and n = 39 for NSE). Adding IL-10 at 6 h to a model containing S100B at 6 h modestly improved discrimination (AUC 0.877 → 0.914; ΔAUC +0.037; LR test *p* = 0.045), and the incremental value over NSE at 48 h was more pronounced (AUC 0.843 → 0.923; ΔAUC +0.080; LR test *p* = 0.001). These findings indicate that early IL-10 captures prognostic information that is partially independent of, and available earlier than, that provided by structural brain injury markers (Table 4).

### 3.6. Penalized Regression and Internal Validation

Because the wide confidence interval for the IL-10 estimate in the maximum-likelihood model raised concern about small-sample instability and possible separation, both multivariable models were re-estimated using Firth’s penalized-likelihood method. The independent association of IL-10 ≥ 154.32 pg/mL with poor outcome was preserved with a more conservative point estimate and a narrower confidence interval (an ~8% reduction) (penalized aOR 10.84, 95% CI 2.00–83.43; *p* = 0.005), as was the protective association of a preserved Th1/Th2 ratio (penalized aOR 0.14, 95% CI 0.02–0.68; *p* = 0.014). Internal validation of the adjusted IL-10 model (age, initial shockable rhythm, and IL-10 ≥ 154.32 pg/mL) by bootstrap resampling (1000 replications, Harrell’s optimism correction, with the Youden cut-off re-derived within each replicate) yielded an apparent AUC of 0.891 and an optimism-corrected AUC of 0.850 (estimated optimism 0.040), with a five-fold cross-validated AUC of 0.832; the single-marker IL-10 discrimination (AUC 0.844) showed negligible optimism. Model calibration was adequate, with a Brier score of 0.118. These modest optimism estimates indicate limited overfitting; nonetheless, the data-derived cut-offs should be regarded as provisional pending external validation.

## 4. Discussion

### 4.1. Principal Findings

In this retrospective analysis of a prospectively enrolled cohort of cardiac arrest survivors treated with TTM, we report three principal findings. First, severe PCAS was characterized by a simultaneous surge of IL-6 and IL-10 peaking at 6 h post-ROSC. Second, IL-10 at 6 h was the most discriminative single cytokine for poor neurological outcome (AUC 0.844), and exceeding 154.32 pg/mL was associated with a 15-fold increase in adjusted odds of poor outcome. Third, an early collapse of the Th1/Th2 balance (IFN-γ/IL-10 ratio < 0.0314) was independently associated with poor outcome, and IL-10 at 6 h added significant incremental information beyond established brain injury biomarkers, S100B and NSE. Because IFN-γ did not differ between outcome groups, this Th1/Th2 observation largely restates the IL-10 finding rather than constituting an independent result.

### 4.2. The Hyperacute 6-Hour Inflection Point

Adrie et al. described PCAS as a sepsis-like syndrome with simultaneous elevations of pro- and anti-inflammatory markers at admission [1]. Bro-Jeppesen et al. extended these observations to TTM-treated patients but evaluated cytokines at 24 h intervals [8,16]. Our study refines this picture by demonstrating that the simultaneous IL-6/IL-10 surge does not merely occur “early,” but reaches its peak at a relatively narrow window approximately 6 h after ROSC. The magnitude of IL-10 elevation at this time point appears to mark the trajectory toward profound immunoparalysis [5,6]. We use the term “immunoparalysis” descriptively, as functional confirmation (e.g., monocytic HLA-DR expression or ex vivo cytokine responses) was not available in this study. This timing is consistent with sepsis literature in which early IL-10 dominance and regulatory T-cell expansion drive subsequent immune dysfunction [6].

### 4.3. Th1/Th2 Imbalance and Early Anti-Inflammatory Dysregulation

The brain–immune axis plays a critical role in secondary neuronal injury after cardiac arrest. Preclinical studies have shown that T lymphocytes infiltrate vulnerable brain parenchyma through a disrupted blood–brain barrier within hours of cerebral ischemia [4]. Th1 cells generally drive cellular immunity and neuroinflammation [17], although early IFN-γ after cardiac arrest is thought to derive more from innate (NK and CD8+) cells than from antigen-specific Th1 responses, whereas Th2 cells and regulatory T cells mediate immunosuppression [18]. Consistent with a predominantly innate rather than antigen-specific source, IL-10 at 6 h was inversely correlated with the circulating monocyte count (Spearman ρ = −0.59, *p* < 0.001). We translated this concept to the clinical setting by examining the IFN-γ/IL-10 ratio as a surrogate of Th1/Th2 polarization. Our finding that an extreme IL-10 dominance over IFN-γ at 6 h was independently associated with poor outcome supports the hypothesis that early Th2/regulatory shift contributes to neuronal injury, possibly by impairing clearance of necrotic debris and increasing susceptibility to nosocomial infection [6,19], although infection rates were not formally analyzed in this dataset. The direction of this association cannot be established from our data; elevated IL-10 may equally reflect, rather than cause, greater injury severity.

### 4.4. Incremental Value over Established Biomarkers

S100B and NSE are the most widely studied serum biomarkers for neuroprognostication after cardiac arrest, but NSE typically reaches its peak prognostic accuracy at 48–72 h, by which time treatment decisions are often already made. Our finding that IL-10 at 6 h adds prognostic information beyond NSE at 48 h, and to a more modest degree beyond S100B at 6 h, is novel and clinically relevant: an immunological biomarker available within the first hours after ROSC could complement structural brain injury markers and potentially enable earlier risk stratification. These results, however, are exploratory and require external validation.

### 4.5. Clinical Implications

Our findings suggest that biomarker-guided risk stratification using a 6 h IL-10 threshold of approximately 150 pg/mL may help identify patients with profound early immunoparalysis. Whether such patients benefit from immunostimulatory rather than immunosuppressive interventions remains an open question; corticosteroid therapy after cardiac arrest, in particular, remains controversial [20]. We caution against direct clinical adoption of the specific cut-off reported here in the absence of external validation, as data-derived thresholds from a single-center cohort of 56 patients are subject to optimism bias.

### 4.6. Limitations

Several limitations should be acknowledged. First, this was a single-center study whose data were collected prospectively but analyzed retrospectively, with a relatively small sample size (n = 56), which limits generalizability and increases the risk of optimistic estimation of cut-offs and effect sizes; to mitigate this we applied Firth’s penalized regression, which stabilized the effect estimates, and quantified overfitting with bootstrap optimism correction, but external validation in independent cohorts remains essential. Second, the cohort included both OHCA and IHCA cases, although sensitivity analysis restricted to OHCA yielded consistent findings. Third, we used circulating cytokines as surrogate markers for T-cell subsets; direct flow cytometric quantification of peripheral T-cell subpopulations would provide more definitive immunological characterization. Fourth, IL-6 values exceeding the upper limit of detection (>10,000 pg/mL) were assigned the upper limit value, which may have biased estimates toward the null; a sensitivity analysis excluding these values yielded a consistent direction of effect. Fifth, multiple comparisons across cytokines and time points were not formally adjusted; primary analyses were pre-specified at 6 h, and other time points should be interpreted as exploratory. Sixth, infection rates and longer-term neurological outcomes (e.g., 90-day or 6-month CPC) were not analyzed in the present report. Finally, target temperature was selected by the treating clinician rather than randomized, which may introduce confounding.

Beyond S100B and NSE, several early blood biomarkers of hypoxic–ischemic brain injury have been proposed for neuroprognostication after cardiac arrest, including serum neurofilament light chain (NfL) [21], total tau [22], and glial fibrillary acidic protein (GFAP) [23]. These structural markers typically reach peak prognostic accuracy at 24–72 h after ROSC, whereas the anti-inflammatory signal described here is already discriminative at 6 h; early IL-10 may therefore complement, rather than replace, later-peaking structural biomarkers within a multimodal prognostic strategy.

## 5. Conclusions

In TTM-treated cardiac arrest survivors, the 6 h post-ROSC window represents a critical inflection point for immune dysregulation. Early elevation of IL-10 and a collapse of the Th1/Th2 balance at 6 h were independently associated with poor neurological outcome at 6 months after cardiac arrest and added incremental information beyond established brain injury biomarkers. External validation in larger, prospective, multicenter cohorts is required before clinical implementation.

## Figures and Tables

**Table 1:** Baseline demographic and clinical characteristics. Data are presented as median (interquartile range) for continuous variables and n (%) for categorical variables. Good outcome was defined as Cerebral Performance Category (CPC) 1–2 and poor outcome as CPC 3–5 at 6 months after cardiac arrest. CPR, cardiopulmonary resuscitation.

Variable	Good Outcome (n = 18)	Poor Outcome (n = 38)	*p*-Value
Age, years	49.0 (39.2–59.2)	66.5 (40.0–73.8)	0.165
Male sex, n (%)	9 (50.0)	25 (65.8)	0.380
Hypertension, n (%)	5 (27.8)	15 (39.5)	0.561
Diabetes mellitus, n (%)	3 (16.7)	9 (23.7)	0.732
Out-of-hospital arrest, n (%)	10 (55.6)	30 (78.9)	0.110
Witnessed arrest, n (%)	5 (27.8)	12 (31.6)	1.000
Bystander CPR, n (%)	13 (72.2)	29 (76.3)	0.751
Initial shockable rhythm, n (%)	12 (66.7)	5 (13.2)	<0.001
Cardiac etiology, n (%)	17 (94.4)	21 (55.3)	0.005
Time to ROSC, min	19.5 (14.0–28.0)	33.0 (24.0–43.5)	0.011
Coronary angiography, n (%)	13 (72.2)	11 (28.9)	0.004
ECPR, n (%)	0 (0.0)	1 (2.6)	>0.99
Vasopressor index at 6 h	0.0 (0.0–4.0)	4.0 (0.0–4.0)	0.090
ICU length of stay, days	6.8 (4.3–8.7)	10.4 (5.5–17.2)	0.067
Monocyte count at 6 h, ×10^3^/µL	6.10 (5.50–7.40)	5.20 (2.70–7.10)	0.096
Target temperature 33 °C, n (%)	14 (77.8)	32 (84.2)	0.706

**Table 2:** Time course of cytokines and the Th1/Th2 ratio after return of spontaneous circulation (ROSC). Data are presented as median (interquartile range) in pg/mL. *p*-values were calculated using the Mann–Whitney U test between the good-outcome (CPC 1–2) and poor-outcome (CPC 3–5) groups. Th1/Th2 ratio = IFN-γ / IL-10 at the same time point.

Biomarker	Time	Good Outcome	Poor Outcome	*p*-Value
IL-6	INIT	152.3 (73.6–292.7)	275.3 (86.1–972.2)	0.297
	6 h	113.8 (49.9–398.7)	596.2 (185.8–3813.5)	0.004
	24 h	46.4 (28.5–121.2)	205.6 (46.9–1005.9)	0.013
	48 h	53.9 (18.7–142.8)	143.8 (42.7–359.6)	0.099
	72 h	49.6 (9.8–315.0)	141.8 (43.6–429.9)	0.150
IL-10	INIT	34.6 (15.4–115.4)	38.5 (15.4–161.6)	0.624
	6 h	57.9 (23.1–125.8)	296.8 (154.3–920.1)	<0.001
	24 h	30.8 (8.4–59.0)	46.1 (25.4–111.7)	0.108
	48 h	27.0 (17.8–37.3)	25.9 (15.2–51.9)	0.992
	72 h	7.6 (3.0–43.9)	37.3 (21.8–71.7)	0.009
IFN-γ	INIT	2.6 (1.0–6.9)	1.9 (0.9–4.2)	0.503
	6 h	2.5 (1.0–13.8)	7.1 (1.3–25.5)	0.403
	24 h	1.6 (0.9–4.4)	1.1 (0.9–5.9)	0.796
	48 h	1.7 (0.9–3.5)	1.0 (0.9–4.0)	0.296
	72 h	1.7 (0.9–4.3)	1.3 (0.9–2.9)	0.382
TNF-α	INIT	49.1 (41.6–60.6)	56.4 (32.5–77.3)	0.868
	6 h	64.7 (43.7–76.2)	88.6 (57.1–134.2)	0.063
	24 h	47.0 (28.2–64.9)	63.1 (35.4–137.7)	0.086
	48 h	41.6 (31.0–59.1)	49.1 (35.6–101.9)	0.374
	72 h	38.2 (31.6–63.9)	65.9 (41.9–99.3)	0.182
Th1/Th2 ratio (IFN-γ/IL-10)	6 h	0.071 (0.036–0.234)	0.009 (0.004–0.053)	0.010
	24 h	0.084 (0.032–0.175)	0.036 (0.018–0.129)	0.214
	48 h	0.060 (0.037–0.130)	0.070 (0.034–0.245)	0.968
	72 h	0.207 (0.049–0.585)	0.040 (0.018–0.462)	0.051

**Table 3:** Multivariable logistic regression for predicting poor neurological outcome (CPC 3–5) at 6 months after cardiac arrest using cut-offs derived from the Youden index applied to 6 h ROC curves. OR, odds ratio; CI, confidence interval.

Model	Variable	Adjusted OR (95% CI)	*p*-Value
Model 1	Age (per 1 year)	1.04 (1.00–1.09)	0.065
	Initial shockable rhythm	0.15 (0.02–1.09)	0.061
	IL-10 ≥ 154.32 pg/mL at 6 h	15.28 (2.02–115.38)	0.008
Model 2	Age (per 1 year)	1.04 (0.99–1.09)	0.092
	Initial shockable rhythm	0.04 (0.01–0.31)	0.002
	Th1/Th2 ratio ≥ 0.0314 at 6 h	0.09 (0.01–0.61)	0.013

**Table 4:** Incremental predictive value of IL-10 at 6 h over established brain injury biomarkers, evaluated by likelihood-ratio (LR) tests of nested logistic regression models for poor neurological outcome (CPC 3–5). IL-10 at 6 h was entered as a log10-transformed continuous variable. Single-marker AUCs are reported for the complete-case subset in which both the reference marker and IL-10 were available (n = 43 for S100B, n = 39 for NSE) and therefore differ from the marginal AUCs shown in Figure 3, which were computed on all patients with the respective marker. AUC, area under the curve; NSE, neuron-specific enolase.

Model	AUC	Δ AUC	LR Test *p*-Value
S100B at 6 h alone (n = 43)	0.877	—	—
S100B at 6 h + IL-10 at 6 h	0.914	+0.037	0.045
NSE at 48 h alone (n = 39)	0.843	—	—
NSE at 48 h + IL-10 at 6 h	0.923	+0.080	0.001

## Data Availability

The data presented in this study are available on request from the corresponding author. The data are not publicly available due to privacy and ethical restrictions.

## Supplementary Materials

The following supporting information can be downloaded at: https://www.mdpi.com/article/10.3390/jcm15166376/s1. Table S1: Sensitivity analyses of the multivariable association between 6-hour cytokine markers and poor neurological outcome at 6 months; Checklist S1: STROBE Statement—checklist of items that should be included in reports of cohort studies.

## Abbreviations

PCAS	post-cardiac arrest syndrome
ROSC	return of spontaneous circulation
TTM	targeted temperature management
OHCA	out-of-hospital cardiac arrest
IHCA	in-hospital cardiac arrest
CPC	Cerebral Performance Category
ROC	receiver operating characteristic
AUC	area under the curve
IL	interleukin
IFN-γ	interferon-gamma
TNF-α	tumor necrosis factor-alpha
NSE	neuron-specific enolase
aOR	adjusted odds ratio
IQR	interquartile range
LR	likelihood ratio
