# Supplementary material for: Hyperacute Cytokine Kinetics and Early Interleukin-10 Elevation as Predictors of Neurological Outcome in Post-Cardiac Arrest Syndrome
Source: J Clin Med. 2026 Aug 18;15(16):6376. doi: 10.3390/jcm15166376 (PMC13513364; doi:10.3390/jcm15166376)
Supplement: Supplementary file 1 [file jcm-15-06376-s001.zip › jcm-4467142-supplementary.pdf]

Table S1. Sensitivity analyses of the multivariable association between 6-hour cytokine markers and poor neurological outcome at 6 months.

Predictor	Primary model, aOR (95% CI), *p*	Additionally adjusted for time to ROSC, aOR (95% CI), *p*
IL-10 ≥ 154.32 pg/mL	15.28 (2.02–115.38), 0.008	8.36 (0.92–75.62), 0.059
Th1/Th2 ratio ≥ 0.0314	0.09 (0.01–0.61), 0.013	0.14 (0.02–1.13), 0.065

All models were fitted by maximum-likelihood logistic regression and adjusted for age and initial shockable rhythm; the second column additionally includes time to ROSC as a covariate. Effect estimates for both predictors retained the same direction after adjustment for time to ROSC, with attenuation to borderline significance consistent with the modest sample size. No IL-6 concentration exceeded the assay upper limit of quantification in this cohort (maximum observed value 8505 pg/mL); the pre-specified sensitivity analysis excluding above-range IL-6 values therefore excluded no participants and yielded results identical to the primary analysis.

aOR, adjusted odds ratio; CI, confidence interval; IL, interleukin; ROSC, return of spontaneous circulation; Th, T-helper.

Checklist S1. STROBE Statement — checklist of items that should be included in reports of cohort studies.

Item	No	Recommendation	Reported in section
**Title and abstract**	1	(a) Indicate the study's design with a commonly used term in the title or the abstract. (b) Provide in the abstract an informative and balanced summary of what was done and what was found.	Title; Abstract
**Introduction**			
Background/rationale	2	Explain the scientific background and rationale for the investigation being reported.	Introduction
Objectives	3	State specific objectives, including any prespecified hypotheses.	Introduction (final paragraph)
**Methods**			
Study design	4	Present key elements of study design early in the paper.	2.1. Study Design and Population
Setting	5	Describe the setting, locations, and	2.1. Study Design

Item	No	Recommendation	Reported in section
		relevant dates, including periods of recruitment, exposure, follow-up, and data collection.	and Population
Participants	6	(a) Give the eligibility criteria, and the sources and methods of selection of participants; describe methods of follow-up. (b) For matched studies, give matching criteria and number of exposed and unexposed.	2.1. Study Design and Population; Figure 1
Variables	7	Clearly define all outcomes, exposures, predictors, potential confounders, and effect modifiers. Give diagnostic criteria, if applicable.	2.4–2.5
Data sources/measurement	8	For each variable of interest, give sources of data and details of methods of assessment (measurement). Describe comparability of assessment methods if there is more than one group.	2.4. Biomarker Measurement; 2.5. Outcome
Bias	9	Describe any efforts to address potential sources of bias.	2.6. Statistical Analysis; Discussion (Limitations)
Study size	10	Explain how the study size was arrived at.	2.1; 2.6
Quantitative variables	11	Explain how quantitative variables were handled in the analyses. If applicable, describe which groupings were chosen and why.	2.6. Statistical Analysis
Statistical methods	12	(a) Describe all statistical methods, including those used to control for confounding. (b) Describe any methods used to examine subgroups and interactions. (c) Explain how missing data were addressed. (d) If applicable, explain how loss to follow-up was addressed. (e) Describe any sensitivity analyses.	2.6. Statistical Analysis
**Results**			
Participants	13	(a) Report numbers of individuals at each stage of study. (b) Give reasons for non-participation at each stage. (c) Consider	3.1; Figure 1

Item	No	Recommendation	Reported in section
		use of a flow diagram.	
Descriptive data	14	(a) Give characteristics of study participants and information on exposures and potential confounders. (b) Indicate number of participants with missing data for each variable of interest. (c) Summarise follow-up time.	3.1; Table 1
Outcome data	15	Report numbers of outcome events or summary measures over time.	3.1; Table 1
Main results	16	(a) Give unadjusted estimates and, if applicable, confounder-adjusted estimates and their precision. (b) Report category boundaries when continuous variables were categorized. (c) If relevant, consider translating estimates of relative risk into absolute risk.	3.3–3.6; Tables 2–4
Other analyses	17	Report other analyses done—e.g., analyses of subgroups and interactions, and sensitivity analyses.	3.6; Table S1
**Discussion**			
Key results	18	Summarise key results with reference to study objectives.	4.1. Principal Findings
Limitations	19	Discuss limitations of the study, taking into account sources of potential bias or imprecision.	Discussion (Limitations)
Interpretation	20	Give a cautious overall interpretation of results considering objectives, limitations, multiplicity of analyses, results from similar studies, and other relevant evidence.	Discussion
Generalisability	21	Discuss the generalisability (external validity) of the study results.	Discussion (Limitations); Conclusions
**Other information**			
Funding	22	Give the source of funding and the role of the funders for the present study and, if applicable, for the original study on which the present article is based.	Funding

An Explanation and Elaboration article discusses each checklist item and gives methodological background and published examples of transparent reporting. The STROBE

checklist is best used in conjunction with this article (freely available at www.annals.org, www.epidem.com, and www.plosmedicine.org).
